# The relationship between anxiety and cardiometabolic risk factors in adolescents with obesity: propensity scores

**DOI:** 10.3389/fendo.2025.1477006

**Published:** 2025-02-06

**Authors:** Miguel Angel Villasis-Keever, Jessie Nallely Zurita-Cruz, Areli Zulema Pichardo-Estrada, Wendy Alejandra Mazón-Aguirre

**Affiliations:** ^1^ Research Unit in Analysis and Synthesis of the Evidence, Hospital de Pediatría, National Medical Center XXI Century, Instituto Mexicano del Seguro Social, Mexico City, Mexico; ^2^ Facultad de Medicina Universidad Nacional Autónoma de Mexico, Hospital Infantil de Mexico Federico Gómez, Mexico City, Mexico

**Keywords:** obesity, anxiety, metabolic syndrome, insulin resistance, cardiometabolic factors

## Abstract

**Background:**

It has been described that there is a relationship between metabolic health and anxiety.

**Objective:**

To determine the relationship between anxiety and metabolic syndrome, as well as cardiometabolic risk factors, in adolescents with obesity.

**Methods:**

We conducted a comparative cross-sectional study of adolescents with obesity between January 2019 and December 2022. In each patient, we recorded somatometric measurements, lipid profiles, and serum insulin levels. Anxiety was measured using the Spence Children’s Anxiety Scale. Participants were divided into those with and without anxiety. Patients with anxiety were matched to patients without anxiety using propensity scores based on z-score body mass index (zBMI). Mann–Whitney U tests and χ^2^ tests were used.

**Results:**

Of the 564 adolescents, 32.6% (n = 184) suffered from anxiety. In the overall study population, no differences in biochemical and cardiometabolic parameters were observed between the adolescents with and without anxiety prior to adjusting the groups based on zBMI. After matching using their zBMI, we found that the adolescents with anxiety had higher serum uric acid levels (5.9 mg/dl vs. 5.4 mg/dl, *p* = 0.041), an increased incidence of metabolic syndrome (39.1% vs. 15.9%, *p* = 0.002), hyperglycemia (21.7% vs. 8.6%, p = 0.020), and lower HDLc (67.3% vs. 34.7%, *p* < 0.001), than those without anxiety. Girls with anxiety had a higher proportion of cardiometabolic risk factors compared to those without anxiety.

**Conclusions:**

Adolescents with obesity and anxiety had higher cardiometabolic risk factors than those without anxiety.

## Introduction

Anxiety disorders are the most common mental health problems among adolescents, with a worldwide prevalence of 6.5% (1). Anxiety disorders typically have their onset during adolescence (2) and are characterized by excessive worry, fear, and apprehension, as well as physical symptoms, such as fatigue, palpitations, and tension (3).

Unlike the many studies that have established a strong association between depression in pediatric patients and being overweight and obese, studies on anxiety are more limited, but research has found an increase in the frequency of anxiety disorders and low self-esteem among children and adolescents with obesity; all of which lead to a deterioration in the quality of life (4, 5). However, it must be considered that it is not clear if being overweight or obese causes anxiety or vice versa. The relationship between anxiety and obesity appears to involve a complex interaction of biological, psychological, and social factors. Biologically, imbalances in appetite-regulating hormones and cortisol have been noted. Psychologically, low self-esteem, negative self-image, and reduced life satisfaction resulting from obesity can contribute to the development of anxiety. Socially, the easy availability of high-calorie fast foods, increased consumption of sugary drinks, extended screen time on electronic devices, and limited opportunities for physical activity are possible contributing factors (6–9).

Moreover, a connection has also been established between anxiety, depression, and a higher risk of cardiovascular disease (CVD). Several pathophysiological factors, including inflammation, oxidative stress, and autonomic dysfunction, have been proposed as systemic processes contributing to this link (10–13). The combined effect of these changes in patients with both obesity and anxiety may accelerate the progression of CVD. In adults with obesity, the metabolic profile tends to be more unfavorable when anxiety is also present (11–14), though similar studies in children and adolescents are limited (15).

The objective of the study was to determine the relationship between anxiety and metabolic syndrome, as well as cardiometabolic risk factors, in adolescents with obesity.

## Methods

### Subjects

This cross-sectional study was conducted in Mexico between January 2019 and May 2022 with a sample of patients from three tertiary care pediatric centers (Hospital Infantil de Mexico Federico Gómez, Pediatric Hospital Centro Médico Nacional Siglo XXI, and High Specialty South Central Hospital of Petroleos Mexicanos). Patients aged 10–18 years with a diagnosis of obesity, defined as a body mass index (BMI) of >95th percentile on the 2000 Center for Disease Control and Prevention (CDC) Growth Charts (16), were included. Exclusion criteria were the presence of genetic syndromes, the use of medications that can influence weight or appetite (e.g., steroids, selective serotonin reuptake inhibitors such as fluoxetine or sertraline, insulin sensitizers, anorexigenics, and intestinal fat absorption inhibitors), the use of hepatotoxic medications, chronic liver disease, and declining the invitation to participate.

### Demographic and clinical information

Demographic information, including age, sex, medical history, and medication use, was collected with the objective of describing the population and identifying whether they met the selection criteria. Anthropometric data, fasting plasma glucose, insulin, and lipid concentrations [high-density lipoprotein cholesterol (HDLc), low-density lipoprotein cholesterol (LDLc), and triglycerides (TGLs)] were collected. Levels of physical sexual maturation were determined by a pediatric endocrinologist based on the Tanner scale, which comprises five stages of pubertal development (17). Children in Tanner stage 1 were classified as prepubertal, Tanner stages 2–4 as pubertal, and Tanner 5 as post-pubescent.

### Anthropometry

A certified nutritionist measured and recorded the anthropometric indicators of each patient. Height was measured using a Seca model 769 stadiometer (Seca GmbH & Co. KG, Hamburg, Germany). Weight measurements were performed using the bioimpedance method (Tanita BC-568 Segmental Body Composition Monitor, Tokyo, Japan). The participants were weighed barefoot in their underwear.

### Anxiety measurement

The presence of elevated levels of anxiety was determined using the Mexican version of the Spence Children’s Anxiety Scale (SCAS) (18). The questionnaire is used as a screening to identify the presence of anxiety. It comprises 38 questions about the respondent’s experience of anxiety symptoms, to which responses are given on a four-point Likert scale with the options never (0), sometimes (1), often (2), or always (3) (19). The SCAS includes six subscales that measure specific anxiety disorders. These are panic attacks and agoraphobia, separation anxiety, social phobia, specific fears, obsessive-compulsive disorder, and generalized anxiety disorder. The Child Report version of the SCAS was used. Elevated anxiety was deemed present when a participant’s total score was ≥60 and a specific anxiety disorder when the score on the relevant subscale was ≥ the 84th percentile. The cut-off scores refer to T-scores to identify children within a subclinical range vs. a clinical range (18).

The global validity and reliability of the SCAS were 0.95 and 0.88, respectively; and in Mexican samples, the validity and reliability were 0.92 and 0.61, respectively (19, 20). The subclinical T-score cut-off (≥60) was used to define the ‘with anxiety’ and ‘without anxiety’ subgroups (18–20).

### Cardiometabolic profile measurement

After a minimum of 12 hours of fasting, blood samples from participants were obtained from the forearm antecubital vein between 7:00 and 8:00 a.m. Serum samples were frozen at −20°C until analysis. Levels of glucose, TGL, HDLc, LDLc, and uric acid were determined using colorimetric enzymatic methods (Bayer Diagnostics, Puteaux, France). Insulin levels were measured by chemiluminescence (Roche/Hitachi Modular P and D Chemistry Analyzer, Roche Diagnostics Corp., Indianapolis, USA; Hitachi Ltd., Tokyo, Japan). Intra- and inter-assay coefficients of variation <7% were considered acceptable. A standard curve was generated for each assay.

### Identification of cardiometabolic health risks

#### Insulin resistance

Each participant’s insulin resistance (IR) index (Homeostatic Model Assessment: HOMA-IR) was calculated using the following formula: HOMA-IR = fasting glucose (mg/dl) X fasting insulin (µU/ml)/405. The HOMA-IR cutoff point for a diagnosis of IR was 2.5 (21).

#### Hypertriglyceridemia

In children <10 years old, hypertriglyceridemia was diagnosed when plasma TGL levels were ≥90th percentile for a child of the participant’s age and sex. In children >10 years old, it was diagnosed when plasma TGL levels were ≥150 mg/dl (22).

#### Altered HDLc and altered LDLc

Low HDLc for children <10 years was judged as that <10th percentile for the participant’s age and sex. In children >10 years, low HDLc was defined as <40 mg/dl in

boys and <50 mg/dl in girls (21). High LDLc was defined as >130 mg/dl (22).

#### Impaired fasting glucose

Elevated fasting plasma glucose was considered a fasting glucose level ≥100 mg/dl (22).

#### Arterial hypertension

Children with hypertension were considered to have diastolic or systolic blood pressure ≥ the 90th percentile for age and sex, according to the National Blood Pressure Education Program Working Group (23).

#### Metabolic syndrome

Metabolic syndrome was defined when at least three of the following cardiometabolic abnormalities were present, according to the definitions already mentioned above: hypertension, obesity, hypertriglyceridemia, reduced HDLc, or elevated fasting plasma glucose (22, 24).

### Statistical analysis

Kolmogorov–Smirnov tests revealed that the quantitative variables had a non-parametric distribution. These were described as the median, minimum, and maximum and the qualitative variables were presented as proportions and frequencies. Comparisons of quantitative variables between groups were performed using the Mann–Whitney U test. For qualitative variables, χ^2^ tests were applied. A *p-*value of <0.05 was considered statistically significant. STATA v.14.0 (Stata Corp. 2015. College Station, TX, USA) was used for the statistical analyses.

### Participant matching

To minimize the impact of any bias introduced by BMI z-scores (zBMI), patients with anxiety were matched to patients without anxiety using propensity scoring. The propensity scores were based on the zBMI. The propensity score technique used was nearest-neighbor matching at a 1:1 ratio without replacement. The caliper was set at 0.01. The pymatch library for Python v.3.7 was used. Subsequently, this analysis was stratified by sex.

### Ethics

This study was conducted in accordance with the tenets of the 2013 version of the Declaration of Helsinki. The protocol was approved by the National Research and Health Ethics Committee of the Mexican Social Security Institute (R-2014-785-024). Both the participants and their parents/caregivers gave written informed consent for participation and publication.

## Results

### Participant characteristics

A total of 589 adolescents with obesity participated in this study. Of these, 25 were excluded due to incomplete questionnaires.

A total of 564 adolescents were analyzed. The sample had a median age of 12 years, with a minimum and maximum of 10 and 18 years, respectively, and there was a predominance of boys (53.6%). The median BMI was 30.1 kg/m^2^ and the median zBMI was 2.4. Of the participants, 92.6% (n = 522) were in Tanner stages 2–4 (pubertal) (**Table 1**).

**Table 1:** General characteristics of the adolescents with obesity with and without anxiety.

Characteristic	Participants, n (%)			*p*
	Total n = 564	No anxiety n = 380	Anxiety n = 184	
Sex, n (%)				0.249
Female	262 (46.4)	187 (49.2)	76 (41.3)	
Male	302 (53.6)	193 (50.8)	108 (58.7)	
Age, years; median (min-max)	12.0 (10.0–18.0)	12.0 (10.0–18.0)	12 (10–18)	0.700
BMI, kg/m^2^; median (min-max)	30.1 (18.5–58.0)	29.5 (10.7–43.2)	30.81 (21.3–58.0)	0.269
BMI z-score, median (min-max)	2.4 (0.8–4.6)	2.43 (1.3–3.6)	2.56 (1.2–4.6)	0.125
Waist circumference, cm, median (min-max)	92.5 (72.0, 117.5)	92.0 (72.0, 116.0)	93.5 (74, 143.4)	0.441
Tanner pubertal stage, n (%)				0.974
1	42 (7.3)	28 (7.3)	14 (7.5)	
2	85 (15.0)	60 (15.7)	25 (13.7)	
3	168 (29.8)	108 (28.5)	60 (32.5)	
4	212 (37.5)	145 (38.2)	67 (36.3)	
5	58 (10.2)	39 (10.3)	18 (10.0)	

min, minimum; max, maximum.

It was noteworthy that the median HDLc was 38.0 mg/dl, which falls below the normal range. The rest of the biochemical parameters had medians that were not significantly different from normal levels for adolescents (**Table 2**). The cardiometabolic parameter that showed the greatest frequency (63.6%, n = 359) of divergence from normal levels was HDLc. Hypertriglyceridemia was found in 41.7% (n = 235) of the sample. IR and metabolic syndrome were identified in 223 patients (39.5%) (**Table 3**).

**Table 2:** Comparison of the biochemical characteristics of adolescents with obesity and with or without anxiety.

Characteristic	Participants, median (min-max)			*p*
	Total n = 564	No anxiety n = 380	Anxiety n = 184	
Biochemical profile, median (min-max)				
Glucose, mg/dl	92.0 (70.0–189.0)	91.4 (70.4–117.0)	92.0 (73.0–124.2)	0.138
HDL cholesterol, mg/dl	38.0 (12.0–65.0)	38.0 (20.0–63.0)	38.0 (12.0–60.0)	0.265
LDL cholesterol, mg/dl	96.0 (56.0–194.0)	96.0 (16.0–194.0)	96.1 (37.4–167.0)	0.717
Triglycerides, mg/dl	140.0 (109.0–533.0)	143.0 (54.0–533.0)	139.5 (40.0–328.0)	0.666
Uric acid, mg/dl	5.8 (0.7–10.0)	5.8 (2.3–10.0)	5.9 (2.0–10.5)	0.302
Insulin, mu/ml	10.9 (2.2–75.2)	10.7 (2.4–79.6)	11.5 (2.2–75.2)	0.481
HOMA-IR	2.4 (0.4–19.2)	2.3 (0.4–19.2)	2.5 (0.4–17.8)	0.377
Systemic blood pressure, median (min-max)				
Systolic, mmHg	114.0 (83.0–146.0)	113.0 (90.0–135.0)	115.0 (88.0–140.0)	0.473
Diastolic, mmHg	71.0 (50.0–100.0)	71.0 (51.0–90.0)	71.0 (50.0–95.0)	0.499

min, minimum; max, maximum.

**Table 3:** Comparison of the cardiometabolic factors of adolescents with obesity and with or without anxiety.

Characteristic	Participants, n (%)			*p*
	Total n =564	No anxiety n = 380	Anxiety n = 184	
Cardiometabolic factors, n (%)				
Impaired fasting glucose	129 (22.9)	76 (20.0)	53 (28.8)	0.126
Altered HDL cholesterol	359 (63.6)	233 (61.3)	127 (69.0)	0.250
Altered LDL cholesterol	51 (9.0)	37 (9.7)	14 (7.6)	0.573
Hypertriglyceridemia	235 (41.7)	161 (42.4)	74 (40.2)	0.712
Arterial hypertension	35 (6.2)	25 (6.58)	9 (4.9)	0.610
Insulin resistance	223 (39.5)	147 (38.7)	76 (41.3)	0.712
Metabolic syndrome	223 (39.5)	145 (38.2)	78 (42.4)	0.517

min, minimum; max, maximum.

### Anxiety-related symptoms

Anxiety-related symptoms were found in 32.6% (n = 184) of the adolescents in this study. Of the six specific disorders identified by the SCAS subscales, separation anxiety disorder occurred most frequently among those with overall anxiety (92.5%, n = 170), followed by panic attacks and agoraphobia (81.0%, n = 149).

In comparing the demographic, biochemical, and cardiometabolic characteristics of adolescents with and without anxiety-related symptoms, we observed non-significant trends indicating higher zBMI (2.6 vs. 2.4, p = 0.125), serum glucose levels (92.0 mg/dl vs. 91.4 mg/dl, p = 0.138) (see **Table 2**), and hyperglycemia (28.8% vs. 20.0%, p = 0.126) among those with anxiety. However, no significant trends were noted for any of the other parameters (**Table 3**).

In view of the tendency toward higher zBMI in adolescents with anxiety, we matched participants from the anxiety and non-anxiety groups based on zBMI. We then compared the lipid profiles and cardiometabolic factors between the groups. This analysis showed that the adolescents with obesity and anxiety had higher serum uric acid levels (5.9 mg/dl vs. 5.4 mg/dl, *p* = 0.041) and lower HDLc levels (37.0 mg/dl vs. 40.0 mg/dl, *p* = 0.019) than those without anxiety. A comparison of cardiometabolic factors found that the adolescents in our sample with anxiety had a significantly higher incidence of hyperglycemia (21.7% vs. 8.6%, *p* = 0.020) and metabolic syndrome (39.1% vs. 15.9%, *p* = 0.002), and significantly lower HDLc (67.3% vs. 34.7%, *p* < 0.001) than those without anxiety (**Table 4**).

**Table 4:** Comparison of the biochemical and cardiometabolic characteristics of adolescents with obesity and with or without anxiety.

Characteristic	Participants		
	No anxiety	Anxiety	
	n = 92	n = 92	*p*
General characteristics, median (min-max)			
BMI z-score	2.6 (1.5–3.3)	2.54 (1.5–3.6)	0.896
Waist circumference, cm	92.5 (74.0-112.5)	93.0 (73.0-121.0)	0.416
Biochemical profile, median (min-max)			
Glucose, mg/dl	90.0 (70.0–108.0)	92.0 (73.0–124.0)	0.059
HDL cholesterol, mg/dl	40.0 (24.0–55.0)	37.0 (16.0–51.0)	**0.019**
LDL cholesterol, mg/dl	91.2 (62.0–145.0)	96 (55.9–155.0)	0.251
Triglycerides, mg/dl	138.0 (64.0–236.0)	128.0 (40.0–328.0)	0.883
Uric acid, mg/dl	5.4 (3.0–8.5)	5.9 (3.7–8.4)	**0.041**
Insulin, mu/ml	12.3 (2.5–79.6)	13.5 (2.2–75.2)	0.394
HOMA-IR	2.7 (0.6–19.2)	2.9 (0.4–17.8)	0.274
Systemic blood pressure, median (min-max)			
Systolic, mmHg	113.0 (90.0–131.0)	115.0 (89.0–139.0)	0.447
Diastolic, mmHg	70.0 (50.0–90.0)	71.0 (50.0–94.0)	0.572
Cardiometabolic factors, n (%)			
Elevated fasting glucose	8 (8.6)	20 (21.7)	**0.020**
Decreased HDL cholesterol	32 (34.7)	62 (67.3)	**<0.001**
Increased LDL cholesterol	2 (2.1)	6 (6.5)	0.404
Hypertriglyceridemia	32 (34.7)	34 (36.9)	0.922
Arterial hypertension	6 (6.5)	6 (6.5)	1.000
Insulin resistance	34 (36.9)	42 (45.6)	0.301
Metabolic syndrome	14 (15.9)	36 (39.1)	**0.002**

min, minimum; max, maximum.

Propensity scoring. Bold values are statistically significant.

Finally, as shown in **Table 5**, when analyzing the data by sex, girls with anxiety exhibited a higher proportion of cardiometabolic risk factors (elevated fasting glucose, decreased HDLc, IR, and metabolic syndrome), compared to their counterparts without anxiety. In contrast, among boys, the only significant finding was a higher proportion of decreased HDLc in those with anxiety compared to those without.

**Table 5:** Comparison of the biochemical and cardiometabolic characteristics of adolescents with obesity and with or without anxiety.

Characteristic	Female, n=101			Male, n=83.			
	No anxiety	Anxiety		No anxiety		Anxiety	*p*
	n = 62	n = 39	*p*	n = 29		n = 54	
General characteristics, median (min-max)							
BMI z-score	2.6 (1.3–3.3)	2.4 (1.2–4.6)	0.301	2.6 (1.6–2.9)		2.6 (1.5–4.1)	0.272
Waist circumference, cm	89.0 (76.6-112.5)	91.5 (73.3-121.0)	0.333	98.0 (85.9-108.9)		94.5 (73.0-117.5)	0.434
Biochemical profile, median (min-max)							
Glucose, mg/dl	88.0 (76.0–97.0)	91.0 (86.0–124.0)	0.069	96.0 (70.0–108.0)		93.0 (74.0–115.0)	0.142
HDL cholesterol, mg/dl	40.0 (24.0–55.0)	37.0 (16.0–59.0)	0.342	42.0 (25.0–54.0)		38.0 (21.0–57.0)	**0.010**
LDL cholesterol, mg/dl	91.2 (62.0–145.0)	99.0 (40.0–146.0)	0.316	90.2 (55.9–122.0)		93.9 (37.4–155.0)	0.175
Triglycerides, mg/dl	134.0 (64.0–236.0)	148.0 (53.0–323.0)	0.294	148.0 (77.0–235.0)		119.0 (40.0–328.0)	0.127
Uric acid, mg/dl	4.8 (3.9–7.1)	5.4 (3.0–8.0)	0.084	5.5 (3.7–8.5)		6.3 (3.3–9.7)	0.590
Insulin, mu/ml	11.4 (4.8–31.5)	16.5 (5.3–40.4)	**0.010**	14.5 (2.5–79.6)		10.5 (2.2–75.2)	0.309
HOMA-IR	2.3 (1.0–7.3)	3.5 (1.3–11.2)	**0.006**	3.5 (0.6–19.2)		2.4 (0.4–17.8)	0.302
Systemic blood pressure, median (min-max)							
Systolic, mmHg	113.0 (90.0–131.0)	115.0 (89.0–139.0)	0.447	112.0 (91.0–130.0)		111.0 (88.0–138.0)	0.347
Diastolic, mmHg	70.0 (50.0–90.0)	71.0 (50.0–94.0)	0.572	71.0 (50.0–92.0)		72.0 (50.0–93.0)	0.572
Cardiometabolic factors, n (%)							
Elevated fasting glucose	0 (0.0)	8 (20.5)	**0.001**	8 (27.6)		13 (24.0)	0.845
Decreased HDL cholesterol	26 (41.9)	28 (71.8)	**0.011**	6 (20.7)		35 (64.8)	**0.001**
Increased LDL cholesterol	3 (4.8)	4 (10.2)	0.298	0 (0.0)		2 (3.7)	0.455
Hypertriglyceridemia	18 (29.0)	19 (48.7)	0.105	13 (44.8)		16 (29.6)	0.224
Arterial hypertension	3 (4.8)	4 (10.2)	0.298	2 (3.7)		1 (1.8)	0.247
Insulin resistance	17 (27.4)	23 (58.9)	**0.007**	17 (58.6)		20 (37.0)	0.102
Metabolic syndrome	4 (6.4)	19 (48.7)	**<0.001**	10 (34.4)		18 (33.3)	0.758

min, minimum; max, maximum.

Propensity scoring, stratified by sex. Bold values are statistically significant.

## Discussion

The primary finding of this study was that 32.5% of the adolescents with obesity also experienced anxiety-related symptoms, with separation anxiety (92.5%) being the most prevalent type of anxiety disorder. Furthermore, adolescents with anxiety demonstrated an increase in cardiometabolic risk factors. Specifically, we observed that these adolescents had higher serum levels of uric acid and glucose, along with lower HDLc, compared to their non-anxious peers. Notably, girls with anxiety exhibited a more adverse cardiometabolic profile. Consistent with our findings, Cheuiche et al. reported a significant association between the severity of anxiety and cardiovascular risk factors, such as larger waist circumference and higher body fat percentage (25).

These findings are novel, especially as pediatric studies on this topic remain limited. For instance, Ji et al. reported that adults with anxiety have a greater risk of metabolic syndrome compared to those without anxiety (15), while van Reedt Dortland et al. found that anxiety and depression are associated with decreased HDLc and increased abdominal obesity (26). Several studies have identified inflammation as a key factor in the development of cardiovascular disease, with a bidirectional relationship to mental health. Anxiety, obesity, and cardiovascular disease are thought to be linked by a complex interaction of biopsychosocial factors and neurobiological mechanisms, such as hormonal imbalances in the hypothalamic-pituitary-adrenal axis and increased cortisol levels (12, 27).

The relationship between fasting hyperglycemia and elevated cortisol is largely attributed to glucocorticoid-induced hepatic gluconeogenesis and impaired insulin secretion, contributing to features of metabolic syndrome (28–30). Impaired insulin function, higher fasting glucose, and increased diabetes risk have also been observed in individuals with anxiety and depression (31). Likewise, in adolescents with obesity, it has been reported that fasting insulin and HOMA-IR levels are 40% higher in those with depression (32).

Another cardiometabolic alteration identified was elevated serum uric acid levels in adolescents with anxiety compared to those without anxiety. This finding is associated with the higher prevalence of metabolic syndrome in adolescents with anxiety. Recent studies have shown that elevated uric acid levels independently predict the development of diabetes and contribute to IR, fatty liver, and dyslipidemia in the context of metabolic syndrome (33). These effects may be driven by mitochondrial oxidative stress and impaired insulin-stimulated nitric oxide production in endothelial cells. Some researchers have also suggested that a high intake of purine- and fructose-rich foods may contribute to elevated uric acid levels, obesity, and the development of metabolic syndrome (34, 35).

Our study indicates that adolescent girls with anxiety are more likely to experience cardiometabolic risk factors compared to their non-anxious peers. Recent research suggests that psychosocial stress might be a more significant risk factor for cardiometabolic disease in women than in men, possibly due to greater exposure to stress or increased susceptibility to its effects (36). Evidence highlights stronger associations between depression, anxiety, and type 2 diabetes in women compared to men (37, 38). Additionally, sex differences have been observed in the relationship between early adversity and obesity, with girls showing a higher risk of developing obesity linked to early-life stress (39). However, recent reviews have pointed out that few studies have explicitly explored sex-related differences in cardiometabolic outcomes (40).

Despite the significant findings, several limitations must be acknowledged. First, the study’s cross-sectional design limits our ability to establish causality between anxiety and cardiometabolic risk factors. Further research is needed to explore the cardiometabolic changes in adolescents with both obesity and anxiety (5). Additionally, it is important to note that we used the SCAS, which is a valid self-report questionnaire that assesses DSM-IV-defined anxiety symptoms in children. Compared to similar tools such as the Screen for Child Anxiety Related Emotional Disorders (SCARED), which correlates well with the SCAS (r = 0.89), the SCAS is shorter and has a simpler factor structure (41, 42). Other widely used instruments, such as the Revised Children’s Manifest Anxiety Scale (43) and the Fear Survey Schedule for Children-Revised (44), are more general measures of anxiety and do not specifically address DSM-IV anxiety disorders. During patient recruitment, the COVID-19 pandemic and associated lockdowns occurred. Most of the sample (76.0%) was collected prior to the pandemic, with patient recruitment temporarily halted during this period and resuming in January 2022 (14.0%, n=137). A sub-analysis comparing patients recruited before and after the pandemic found no significant differences in the proportion of anxiety. This may be attributed to the fact that the latter group of patients was no longer experiencing social isolation at the time of their inclusion in the study.

As a final reflection, we would like to discuss how to incorporate the study findings into the management of obesity in adolescents. Latin America and Mexico are experiencing an epidemiological transition, with rising rates of childhood obesity and chronic diseases that increase morbidity and mortality (45). Furthermore, psychological changes during adolescence may exacerbate the negative emotions associated with obesity, creating a vicious cycle. Based on the above, it seems important that weight reduction interventions should incorporate mental health strategies (such as relaxation techniques, meditation, and cognitive-behavioral therapy) to enhance adherence to weight reduction programs and improve both short- and long-term health outcomes (46, 47).

## Conclusions

We found that adolescents with obesity and anxiety had higher serum uric acid levels, lower HDLc levels, and higher incidences of hyperglycemia and metabolic syndrome than adolescents with obesity but without anxiety. It is of the utmost importance to develop a multidisciplinary treatment for this population that considers nutritional advice support, teaches coping skills, encourages meditation, and provides cognitive-behavioral therapy.

## Data Availability

The raw data supporting the conclusions of this article will be made available by the authors, without undue reservation.
